# Development and Evaluation of Novel Statistical Methods in Urine Biomarker-Based Hepatocellular Carcinoma Screening

**DOI:** 10.1038/s41598-018-21922-9

**Published:** 2018-02-28

**Authors:** Jeremy Wang, Surbhi Jain, Dion Chen, Wei Song, Chi-Tan Hu, Ying-Hsiu Su

**Affiliations:** 1 JBS Science, Inc., Doylestown, Pennsylvania United States; 2 ClinPharma Consulting, Inc, Phoenixville, Pennsylvania United States; 3 Buddhist Tzu Chi General Hospital and Tzu Chi University, Hualien, 970 Taiwan R.O.C.; 4 The Baruch S. Blumberg Institute, Doylestown, Pennsylvania United States

## Abstract

Hepatocellular carcinoma is one of the fastest growing cancers in the US and has a low survival rate, partly due to difficulties in early detection. Because of HCC’s high heterogeneity, it has been suggested that multiple biomarkers would be needed to develop a sensitive HCC screening test. This study applied random forest (RF), a machine learning technique, and proposed two novel models, fixed sequential (FS) and two-step (TS), for comparison with two commonly used statistical techniques, logistic regression (LR) and classification and regression trees (CART), in combining multiple urine DNA biomarkers for HCC screening using biomarker values obtained from 137 HCC and 431 non-HCC (224 hepatitis and 207 cirrhosis) subjects. The sensitivity, specificity, area under the receiver operating curve, and variability were estimated through repeated 10-fold cross-validation to compare the models’ performances in accuracy and robustness. We show that RF and TS have higher accuracy and stability; specifically, they reach 90% specificity and 86%/87% sensitivity respectively along with 15% higher sensitivity and 10% higher specificity than LR in cross-validation. The potential of RF and TS to develop a panel of multiple biomarkers and the possibility for self-training, cloud-based models for HCC screening are discussed.

## Introduction

Liver cancer is one of the fastest growing cancers in the US and is the sixth most diagnosed and second deadliest cancer in the world, leading to over 745,000 deaths a year^1,2^; 75–90% of cases are hepatocellular carcinoma (HCC)^3^. The current most-used biomarker for HCC screening is serum alpha fetoprotein (AFP), which has been shown to possess limited sensitivity (~40–60%)^4^ at the cutoff of 20 ng/mL recommended by American Association for the Study of Liver Diseases (AASLD)^5^. Due to the lack of efficient screening methods, many cases of HCC persist undiagnosed until later stages, when the 5-year relative survival rate is only 18%^2^. However, the survival rate can be as high as 35% if HCC is detected early; thus, early detection is critical for improving the prognosis of HCC^5^.

Multiple noninvasive biomarkers, including both genetic markers (mutations of *TP53 249* *T*) and epigenetic markers [aberrant methylation of *RASSF1A (mRASSF1A)* and *GSTP1 (mGSTP1)* genes] have been reported to have potential in detecting HCC^6–15^. It has been suggested that a panel of multiple biomarkers derived from different cancer pathways is needed to account for the heterogeneity inherent to HCC^16^ and attain a sufficiently sensitive and robust screening test^17–19^. These three markers (*TP53 249* *T*, *mRASSF1A*, and *mGSTP1*) were chosen for development for HCC screening because they were shown to be detectable in the urine (a preferable medium) of patients with HCC. More importantly, they are derived from different cancer pathways, thus providing the potential to overcome the high heterogeneity of HCC^9^.

To analyze multiple variables and generate algorithms for classification, many different multivariate models can be applied (e.g. k-nearest neighbor and Bayesian classifiers, etc)^20,21^. Among these, logistic regression (LR) is very commonly used, and classification and regression trees (CART) have also become popular. However, LR and CART may lack the robustness necessary to serve as effective algorithms for cancer screening because the cancer’s heterogeneity results in substantial variation in the types (quality) and amount (quantity) of biomarkers across populations. One of the primary goals of this study was therefore to identify and develop a model that would be able to achieve high sensitivity while maintaining robustness.

Machine learning techniques have been used in the field of classification, showing promise in predictive accuracy and robustness in various heterogeneous classification settings, e.g. the human gut microbiome and detection of cancers such as ovarian, lung and breast^22,23^. Although random forest (RF) has been useful in selection of genetic features for HCC detection^24^, to our knowledge, RF has not yet been used to build an HCC screening or diagnosis test using multiple biomarkers. Because of RF’s strength in biological applications of machine learning (e.g. gene ranking, discovery, and selection as well as cancer classification)^25^, it was expected that RF would be a useful tool to build a powerful classification algorithm with multiple biomarkers in HCC screening, and that the ensemble learning in RF would also provide potential to reduce over-fitting with respect to CART as well as additional benefits such as being non-parametric, reasonably efficient, and highly accurate^26^. In addition, we took advantage of AFP’s high specificity (>90%) at the 20 ng/mL cut-off, to propose the fixed sequential (FS) algorithm, which uses AFP and LR in successive splits, potentially leading to improved performance. By introducing a univariate split based on AFP, which is relatively stable across populations, it could also be expected to increase the robustness of the overall model.

Another model, two-step (TS) has been developed by combining traditional statistical modeling with RF using the FS model approach with two multivariate splits. The first split is based on a logistic regression model with a high specificity cutoff and the second uses a random forest trained for further classification. By utilizing two different multivariate models in this novel TS model, we aimed to further increase accuracy.

In summary, this study used biomarker values obtained from the study cohort of 137 HCC and 431 non-HCC (224 hepatitis and 207 cirrhosis) to compare five multivariate models, LR, CART, RF, FS, and TS, based on robustness and predictive accuracy and identify the best model for developing multiple biomarkers into a single panel for use as a sensitive HCC screening test. Model performance was evaluated based on sensitivity, specificity, and area under receiver operating characteristic curve (AUC) using repeated 10-fold cross validation (1,000 iterations). Robustness was examined via variation in the validation data of each iteration. The results from both model building and cross-validation datasets suggest that TS and RF improve upon AFP, LR, CART, and FS, in developing four genetic and epigenetic biomarkers into a potentially robust and sensitive HCC screening test.

## Results

### Summary Statistics and Univariate Analysis in the Full Dataset

The demographic characteristics of the study subjects, 137 HCC and 431 non-HCC (224 hepatitis and 207 cirrhosis), in the dataset are summarized in Table 1. The missing data for each demographic factor is noted as well. The mean and median age are reported along with standard deviation and range, and percentage of males or HBV/HCV positive subjects is also provided. As shown, the differences in demographic values (i.e. age, gender, HBV, and HCV) are of mixed significance [p < 0.0001 (t-test), 0.1315 (chi-squared test), <0.0001 (chi-squared test), 0.0604 (chi-squared test), respectively].

**Table 1:** Study population.

Variables		HCC (n = 137)	Non-HCC (n = 431)	p-value
Age (years)	n (missing 55)	137	376	<0.0001
	Mean (SD)	61.3 (11.4)	55.3 (10.7)	
	Median (Range)	61.0 (26.0–88.0)	55.5 (24.0–81.0)	
Gender	n (missing 3)	137	428	0.1315
	Male: n (%)	107 (78%)	304 (71%)	
HBV	n (missing 46)	129	393	<0.0001
	Positive: n (%)	75 (58%)	308 (78%)	
HCV	n (missing 51)	112	405	0.0604
	Positive: n (%)	37 (33%)	96 (31%)	

**Figure 1:**
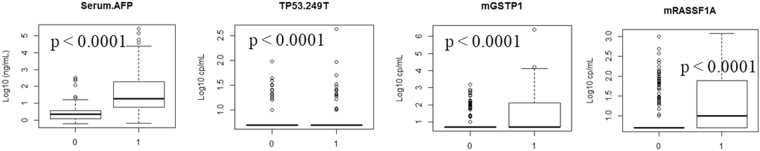
Box plot of each biomarker in HCC and Non-HCC. Each biomarker value was plotted by disease group. “0” indicates non-HCC (n = 431) and “1” indicates HCC (n = 137). P-values were generated via Wilcoxon rank sum test.

Box plots were generated to assess each urine biomarker in relationship with HCC status. Serum AFP was also analyzed as a control for current HCC biomarkers. As shown in Fig. 1, serum AFP as well as urine *mRASSF1A*, *mGSTP1*, and *TP53 249* *T* had statistically significantly higher levels in the HCC group as compared to the non-HCC group (Wilcoxon rank sum test p < 0.0001 for all 4 biomarkers).

Correlation analysis was performed on the biomarkers and demographics (age and gender) to examine whether improvements in screening performance were due primarily to the combination of biomarkers or confounded with demographics. There was evidence of only weak correlations between the biomarkers and age/gender (Table 2), suggesting that the combination of biomarkers most likely accounted for improvements to screening performance.

**Table 2:** Summary of relationships between demographics and biomarkers.

Correlation Coefficients		
	Age (Spearson’s ρ, n = 513)	Gender (Point Biserial w/ Log Transform, n = 565)
TP53.249 T	0.125	−0.144
mRASSF1A	0.143	−0.058
mGSTP1	0.107	0.007
Serum.AFP	0.137	−0.055

**Figure 2:**
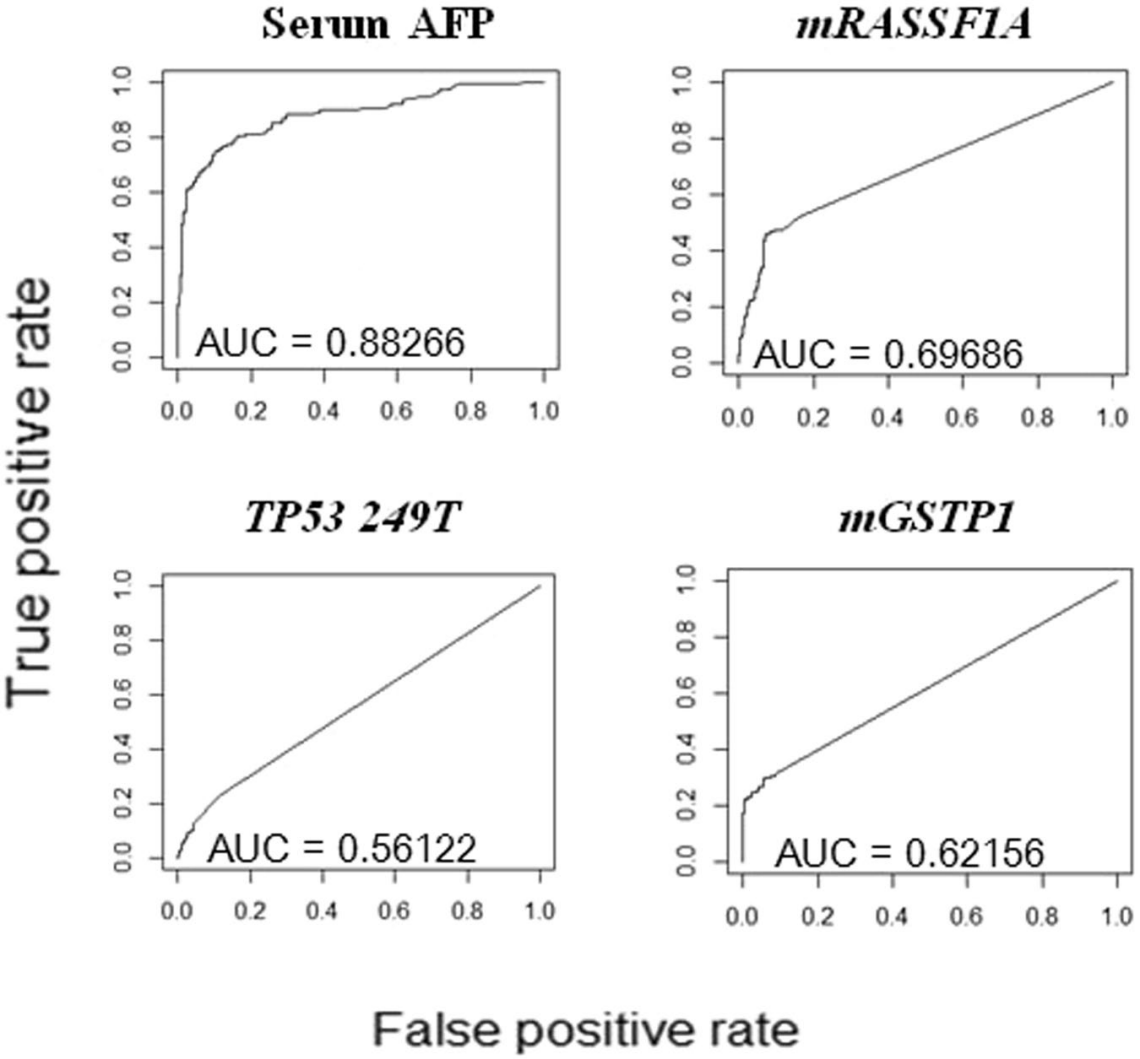
Performance of each biomarker in HCC classification as evaluated by Univariate ROC curves and AUC (n = 568; HCC 137, Non-HCC 431).

Next, to evaluate the performance of each marker in distinguishing HCC from non-HCC, a univariate logistic regression model was generated for each individual biomarker on the entire dataset. Receiver operating characteristic (ROC) curves were generated and AUCs were calculated for each of the biomarkers (Fig. 2). In this study, none of the urine biomarkers performed better individually than serum AFP (AUC = 0.88) in terms of AUC, with AUC ranging from 0.56 to 0.70.

### Multivariate Analysis in the Full Dataset

As mentioned, multiple markers from multiple cancer pathways are likely needed to improve the sensitivity of HCC screening. To compare the accuracy and robustness of the proposed multivariate models FS, RF and TS with those of LR and CART in HCC classification, 1,000 iterations of 10-fold cross-validation were generated. AUC, sensitivity, and specificity were used to compare the predictive accuracy of the models while the robustness was evaluated through the variability of model results in the validation data.

**Figure 3:**
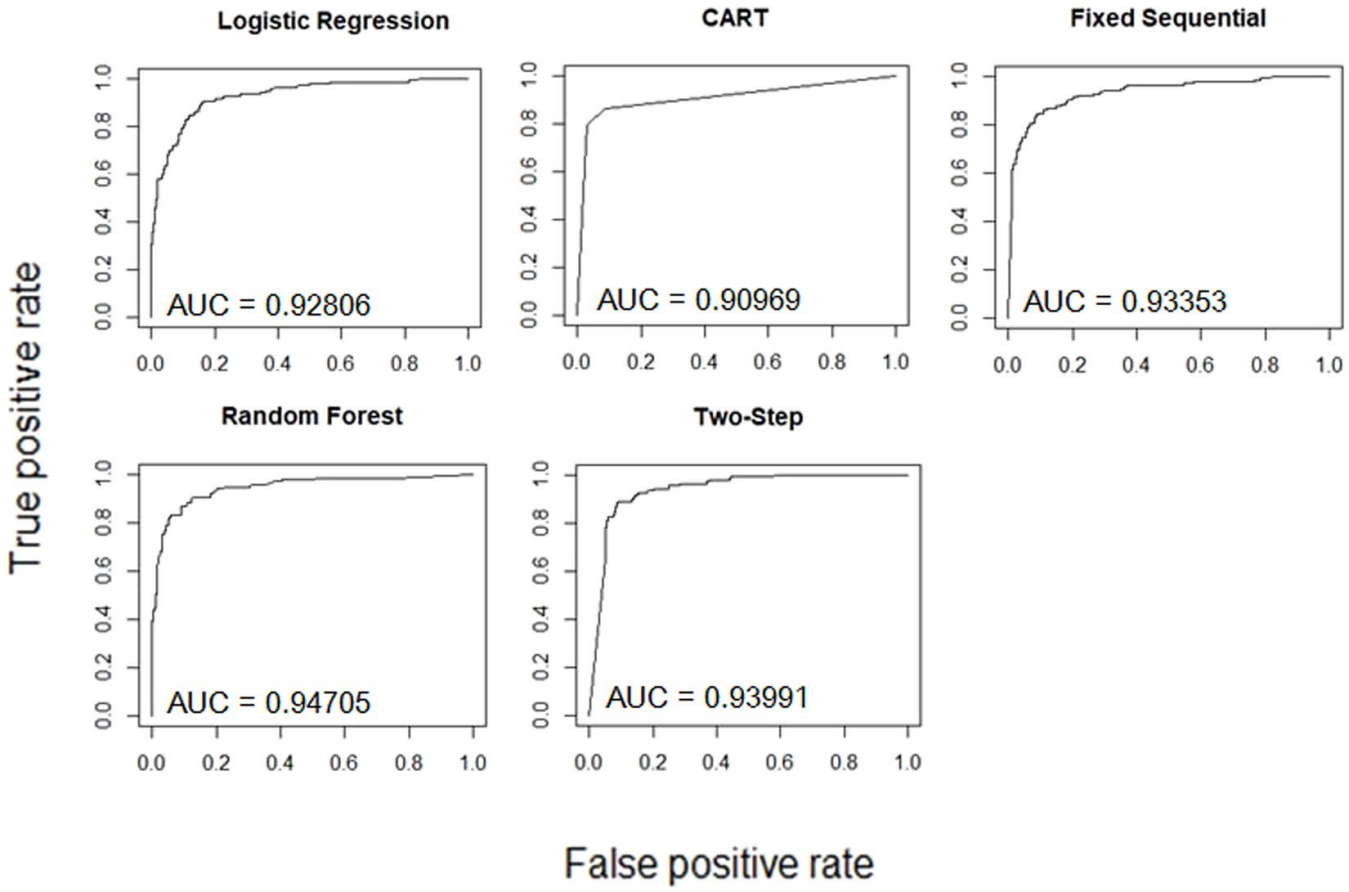
ROC curves and AUCs generated using multivariate models LR, CART, FS, RF, and TS for distinguishing HCC (n = 137) from non-HCC (n = 431).

ROC curves were constructed using all five models on the full dataset of 586 subjects as shown in Fig. 3, and the AUCs of each cross-validation iteration are calculated and summarized in Table 3. As expected, each of the five multivariate models (AUC ranging from 0.91 to 0.95) performed better in terms of AUC than the current most used biomarker, serum AFP (AUC = 0.88). Among the five models, RF and TS performed better than LR, CART and FS based on mean AUCs.

**Table 3:** Summary of multivariate models’ AUCs from cross-validation.

AUC	Model Building		Cross Validation	
	Mean (95% CI)	Median (Range)	Mean (95% CI)	Median (Range)
LR	0.928 (0.927–0.928)	0.928 (0.927–0.929)	0.926 (0.921–0.931)	0.926 (0.915–0.934)
CART	0.910 (0.909–0.912)	0.910 (0.905–0.914)	0.897 (0.886–0.908)	0.897 (0.856–0.910)
FS	0.934 (0.934–0.934)	0.934 (0.933–0.934)	0.933 (0.928–0.937)	0.932 (0.925–0.940)
RF	0.950 (0.949–0.951)	0.950 (0.948–0.952)	0.938 (0.932–0.945)	0.938 (0.927–0.949)
TS	0.945 (0.944–0.946)	0.945 (0.943–0.947)	0.935 (0.930–0.940)	0.935 (0.923–0.946)

### AUC

The mean AUCs for RF and TS were higher than those of LR, FS, and CART in the model building set. Notably, TS’ lower AUC when compared to that of RF is due in most part to the slope in the beginning of the curve; however, at lower specificity, TS attained a similar sensitivity to RF.

From the model building to the validation set, RF and TS’ AUC values decreased by approximately 1% in both the mean and median, while the FS and LR models retained essentially the same AUC. Specifically, in the validation set, RF and TS reached AUCs of approximately 0.94 and the LR and FS models achieved slightly smaller AUCs of approximately 0.93, while CART had the smallest AUC of 0.91. These four models were all similarly robust in terms of AUC, with very little variance (maximum error margin of ± 1%) and a tight range (at most 5.4%). Thus, the TS and RF models are shown to perform similarly well in AUC and consistently better than FS, LR, and CART in both model building and validation sets.

### Sensitivity and Specificity

Next, we compared the sensitivities of the five multivariate models at pre-fixed specificities (Table 4). Of the five models, TS generally performed the best, especially at higher, more restrictive cutoffs of specificity. In the validation set, TS reached up to 15% higher sensitivity than LR and 3.2% higher sensitivity than the next most sensitive model, RF. At a cutoff of 90% specificity, TS achieved approximately 87% sensitivity, a substantial improvement over both AFP (48.2% sensitivity in this population) and CART/LR (74%/76% sensitivity, respectively).

**Table 4:** Summary of multivariate models’ sensitivities at different cutoffs of specificity from the 1,000 iterations of 10-fold CV for both model building and validation data.

Specificity Cutoff	Model	Model Building		Cross Validation	
		Mean (95% CI)	Median (Range)	Mean (95% CI)	Median (Range)
85%	LR	0.873 (0.869–0.878)	0.873 (0.865–0.882)	0.870 (0.853–0.886)	0.869 (0.842–0.898)
	CART	0.861 (0.857–0.864)	0.861 (0.849–0.868)	0.766 (0.738–0.795)	0.766 (0.667–0.798)
	FS	0.873 (0.870–0.875)	0.873 (0.870–0.877)	0.871 (0.863–0.879)	0.869 (0.860–0.885)
	RF	0.910 (0.907–0.913)	0.910 (0.904–0.915)	0.900 (0.889–0.910)	0.898 (0.882–0.914)
	TS	0.915 (0.913–0.917)	0.915 (0.912–0.918)	0.906 (0.894–0.919)	0.906 (0.884–0.920)
90%	LR	0.789 (0.781–0.797)	0.789 (0.777–0.840)	0.782 (0.757–0.807)	0.781 (0.743–0.820)
	CART	0.861 (0.856–0.865)	0.861 (0.843–0.865)	0.766 (0.738–0.795)	0.766 (0.667–0.798)
	FS	0.846 (0.843–0.849)	0.846 (0.840–0.851)	0.838 (0.826–0.851)	0.839 (0.823–0.863)
	RF	0.870 (0.866–0.874)	0.870 (0.862–0.877)	0.862 (0.848–0.876)	0.862 (0.831–0.877)
	TS	0.890 (0.883–0.891)	0.887 (0.880–0.893)	0.871 (0.857–0.885)	0.870 (0.837–0.899)
95%	LR	0.655 (0.647–0.662)	0.655 (0.643–0.668)	0.645 (0.622–0.669)	0.644 (0.610–0.686)
	CART	0.809 (0.793–0.825)	0.809 (0.780–0.829)	0.664 (0.571–0.757)	0.664 (0.481–0.734)
	FS	0.750 (0.745–0.753)	0.749 (0.745–0.758)	0.738 (0.723–0.752)	0.737 (0.715–0.760)
	RF	0.790 (0.781–0.799)	0.790 (0.776–0.809)	0.766 (0.746–0.786)	0.766 (0.727–0.797)
	TS	0.827 (0.818–0.835)	0.827 (0.811–0.840)	0.798 (0.775–0.822)	0.800 (0.750–0.831)

The results of specificity analysis are similar to the results of sensitivity (Table 5). As expected, the RF and TS models perform better than LR and CART. Interestingly, CART has extremely low specificity at all cutoffs of sensitivity due to the fact that its limited number of predicted probabilities provides no cutoff that can achieve 90% or higher sensitivity while also being high enough to restrain the false positive rate.

**Table 5:** Summary of multivariate models’ specificities at different cutoffs of sensitivity from the 1,000 iterations of 10-fold CV for both model building and validation data.

Sensitivity Cutoff	Model	Model Building		Cross Validation	
		Mean (95% CI)	Median (Range)	Mean (95% CI)	Median (Range)
90%	LR	0.823 (0.816–0.830)	0.823 (0.812–0.832)	0.823 (0.811–0.836)	0.824 (0.800–0.842)
	CART	0.01 (0.000–0.049)	0.01 (0.000–0.102)	0.005 (0.903–0.920)	0.005 (0.893–0.928)
	FS	0.812 (0.809–0.816)	0.812 (0.807–0.820)	0.812 (0.803–0.821)	0.812 (0.798–0.828)
	RF	0.867 (0.861–0.874)	0.867 (0.857–0.876)	0.856 (0.844–0.869)	0.856 (0.833–0.873)
	TS	0.888 (0.884–0.893)	0.888 (0.881–0.896)	0.879 (0.869–0.889)	0.879 (0.856–0.900)
95%	LR	0.640 (0.631–0.648)	0.639 (0.630–0.654)	0640 (0.627–0.652)	0.638 (0.622–0.673)
	CART	0.01 (0.000–0.011)	0.01 (0.000–0.014)	0.005 (0.901–0.923)	0.005 (0.884–0.928)
	FS	0.660 (0.651–0.670)	0.660 (0.644–0.680)	0.659 (0.644–0.674)	0.659 (0.633–0.687)
	RF	0.738 (0.727–0.749)	0.738 (0.716–0.752)	0.724 (0.705–0.742)	0.724 (0.692–0.752)
	TS	0.739 (0.730–0.749)	0.739 (0.725–0.758)	0.730 (0.713–0.746)	0.729 (0.698–0.759)
99%	LR	0.233 (0.217–0.250)	0.234 (0.212–0.253)	0.233 (0.210–0.256)	0.232 (0.204–0.281)
	CART	0.01 (0.000–0.011)	0.01 (0.000–0.014)	0.005 (0.901–0.923)	0.005 (0.884–0.928)
	FS	0.223 (0.206–0.239)	0.218 (0.216–0.259)	0.223 (0.204–0.241)	0.220 (0.211–0.269)
	RF	0.365 (0.293–0.438)	0.367 (0.218–0.455)	0.356 (0.279–0.433)	0.357 (0.197–0.459)
	TS	0.271 (0.186–0.356)	0.269 (0.132–0.451)	0.263 (0.174–0.351)	0.263 (0.125–0.450)

In the validation set, RF and TS are much better than LR, CART, and FS, and at a cutoff of 95% sensitivity, RF and TS reach almost 10% higher specificity (73% compared to 64%). Sensitivities and specificities of the models at their respective cutoffs are available in full in Supplementary Table S1.

In comparison of both sensitivity and specificity, we also compared the performance between the model building and validation data. LR and FS remained stable across the two datasets, but RF/TS weakened slightly (at most 3%). CART exhibited the greatest drop in performance when examined in the validation set.

In this dataset, the relatively narrow confidence intervals and ranges of sensitivity and specificity were noted for all four models compared. For example, TS’ sensitivity at 90% specificity had a 95% confidence interval of 0.857–0.885 in the validation set, and RF had a range of 0.833–0.873 in specificity at 90% sensitivity.

In summary, the cross-validation results are consistent with the model building data AUCs and show that a panel of multiple markers performed better than the current most used marker, serum AFP (sensitivity: 48.2%; specificity: 99%), for detecting HCC. Furthermore, RF and TS performed substantially better than CART and LR in sensitivity and specificity.

## Discussion

This study applied the machine learning algorithm, random forest (RF), proposed the novel statistical algorithms fixed sequential (FS) and two-step (TS), and compared them with both the commonly used multivariate techniques logistic regression (LR) and classification/regression trees (CART), as well as with each other, as models for the development of a sensitive and robust HCC screening test using multiple biomarkers. The algorithms (FS and TS) developed in our study comprised AFP and three urine DNA markers and achieved up to 87% sensitivity and 90% specificity in the validation set, while AFP alone achieved 99% specificity but only 48.2% sensitivity based on the cutoff of 20 ng/mL as recommended by AASLD. Furthermore, these three models provided a substantial improvement (up to 15% higher sensitivity and 10% higher specificity than LR in cross-validation) in performance over the commonly used models LR and CART within the iterative cross-validation experiment. Results from the 10-fold cross-validation demonstrated that RF and TS provide more robust and effective algorithms than either LR or CART for the prediction of HCC using multiple markers.

When comparing the performances of the models across the model building and validation sets, we found that LR and FS remained stable and RF and TS exhibited only slight drops in accuracy, with only CART showing any substantial decrease in performance. CART’s lower performance is likely due to potential misrepresentation of the population in any of the parent nodes causing prediction errors downstream^27^. Thus, we conclude that over fitting was not a concern in this study. However, while it was not found to be a major problem in this study, we anticipate that RF and TS may be able to partially resolve over fitting in other, higher dimensional data when it becomes of greater concern.

Given the significance of age and viral etiology to HCC incidence, addition of clinical variables would likely improve the performance of the models, but the current data is missing these values on certain subjects, prohibiting their addition.

To our knowledge, this is the first time the machine learning technique random forest has been used for modeling multiple biomarkers in HCC screening. Various other machine learning techniques such as neural networks and support vector machines^28^ have been employed in biomarker development, so it would be of interest to examine their performance in this setting. It would also be valuable to investigate the performances of these classification models in other cancers as well.

In conclusion, we demonstrated the potential of machine learning and novel statistical approaches, the fixed sequential model and the two-step model, in aggregating multiple liver cancer biomarkers for developing a highly sensitive HCC screening test. The prediction model combines AFP and three urine DNA markers using the two-step model to reach approximately 87% sensitivity at reasonable specificity with high robustness, thus increasing sensitivity by almost 40% compared to the current most-used method, the serum AFP test, as well as by approximately 9% over the commonly-used multivariate logistic regression model.

Random forest’s and, by extension, two-step’s unique characteristics as an ensemble-type machine learning method may also provide further benefit in future research by enabling them to scale well with large data sets. We envision a cloud-based algorithm that gathers data inputted by users into a single master data set for analysis. As the data set increases in size and dimensionality, a random forest algorithm or other machine learning technique would retrain regularly, incorporating the new data and periodically updating the available model. This would yield an artificially intelligent and dynamically modifiable model that could be highly accurate and robust, making it capable of detecting more cases of HCC earlier, thus improving HCC prognosis.

## Methods

### Subjects, Biomarkers and Data Organization

Urine samples were either obtained from Buddhist Tzu Chi General Hospital with written informed consent and under institutional review board approvals from the Research Ethics Committee of the Hualien Tzu Chi Hospital, Buddhist Tzu Chi Medical Foundation in Hualien, Taiwan or from archived samples as described previously^10^. All experiments were performed in accordance with relevant guidelines and regulations. Our model building dataset included 568 total subjects, 137 of which were HCC patients and 431 were non-HCC (207 cirrhosis and 224 hepatitis). Data is included in Supplementary Table S3. The three biomarkers, excepting serum AFP, are genetic mutations (*TP53 249 T*) and epigenetic methylation markers [aberrant methylation of the *RASSF1A (mRASSF1A)* and *GSTP1 (mGSTP1)* genes] that are detectable in urine of patients^7,9–12^. The biomarkers were quantified as detailed previously^12^.

### Statistical Methods

#### Descriptive Statistics and Univariate Analysis

Summary statistics were generated for both biomarkers and demographic values (e.g. age, gender, and viral etiology). Biomarkers were compared visually via box plots and statistically via Wilcoxon rank sum tests, and demographic values via t-tests and chi-square tests in order to examine their significance to the incidence of HCC. The relationships between demographics (age and gender) and biomarkers were examined using correlation analysis, Spearman’s correlation for age and point biserial correlation with log-transformed biomarker values for gender. Univariate logistic regression was applied to each biomarker, and ROC curves for each biomarker were generated and AUCs were calculated.

### Multivariate Models

LR, CART, FS, RF, and TS were compared as methods for the combination of the examined biomarkers using predictive accuracy and robustness. They were implemented using R^29^.

### Logistic Regression

The full logistic regression (FLR) is a special case of generalized linear model; its primary goal is to predict a binary outcome based on a set of categorical or continuous predictor variables^30^. This study implemented LR using the binomial family of the “glm” function in R with all parameters left at the default. The ROCR package was used to retrieve the performance characteristics of the LR model^31^.

### Classification and Regression Trees

Classification and regression trees are recursively partitioned models that utilize a series of univariate splits to divide data into nodes with particular classifications or probability values^32^. Regression trees were implemented via the rpart function in the “rpart” package in R with *method* set to “class” and all other parameters and controls left at default. Pruning was performed on the generated tree using the prune function in the “rpart” package^33^ and the complexity was set to the value that gave the minimum xerror. Again, the ROCR package was used to retrieve the performance characteristics of the model^31^.

### Fixed Sequential Model

**Figure 4:**
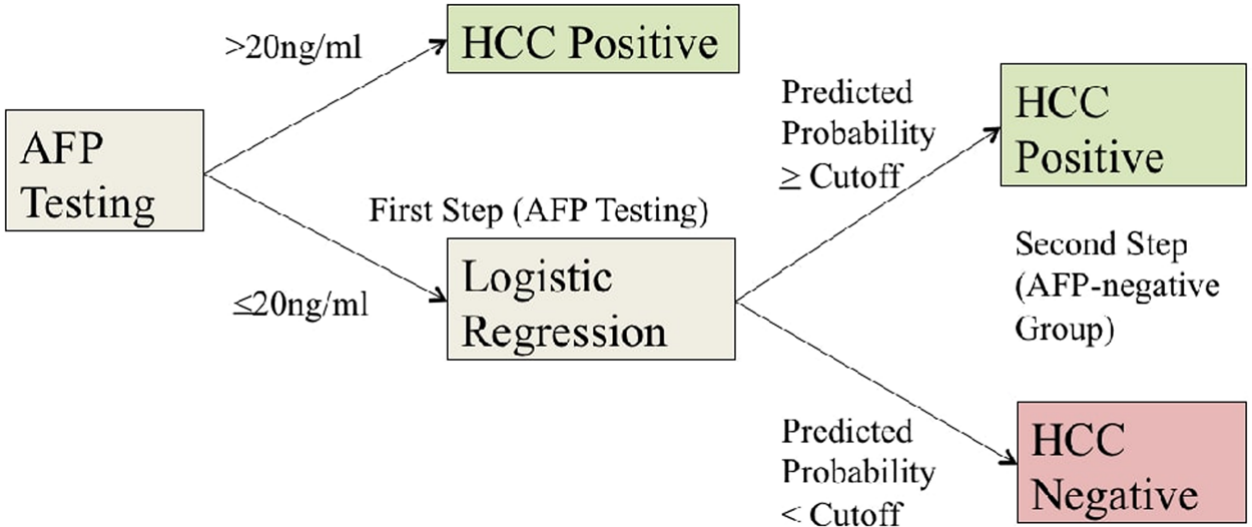
Fixed Sequential Flowchart Data are first separated based on the AASLD standard cutoff of 20 ng/mL. The AFP-positive subjects are predicted as HCC-positive (p = 1), while the AFP-negative subset is run through a logistic regression algorithm that provides a final classification.

The fixed sequential model (FS) involves two steps (Fig. 4). The first step splits the subjects based on serum AFP levels with a cutoff of 20 ng/mL, which is suggested by the American Association for the Study of Liver Disease (AASLD) and produces a high specificity of 90%^5^. The model then trains a logistic regression model (using the same function and parameters as specified above) on and applies logistic regression diagnosis (including all four biomarkers) to the subset of cases that were AFP-negative (i.e., AFP < 20 ng/mL). In order to generate ROC curves, all subjects that were AFP-positive were assigned a predicted probability of one, while the predicted probabilities of AFP-negative subjects were those generated by the logistic regression model.

### Random Forest

Random forest, a non-parametric, supervised machine-learning approach, combines bootstrap aggregation and decision trees in order to maximize the robustness of the model. In RF, subsets of the data are randomly selected with replacement. This data is then divided again, with approximately one-third of the data being left out as out-of-bag (OOB) data. A tree is then constructed on the remaining two-thirds of data. These trees are developed in the same manner as a standard classification tree; the data is separated into various terminal nodes based on univariate splits. Upon completion, this forest of trees may be applied to diagnosis by running cases through each tree in the forest. At the end of the run, the observation weights of the trees will be averaged based on their respective weights to produce a final result. The function ‘cforest’ in the package “party” was used to implement conditional inference random forests in R^34–36^. *Ntree* was set to 500, and the random forest was controlled with a set number of randomly preselected variables (*mtry* = $\surd 3$). All other hyper parameters in the ‘cforest_control’ object were left at the default.

### Two Step Model

The fourth method evaluated in this study is the Two-Step (TS) model, an improved version of FS that integrates LR and RF. TS improves upon both steps of FS by replacing AFP with a more specific classifier (LR at 95% specificity) in the first split, and LR with a more sensitive classifier (RF using the same function and parameters as specified above) in the second split. In addition, LR and RF provide unique perspectives; that is, due to their different structures, each has the potential to correct the inaccuracies of the other.

### Cutoff Points

Various points of both sensitivity and specificity were tested to have a series of constant points of comparison among the four models. Accordingly, cutoff points were set at 85%, 90%, and 95% specificity to evaluate sensitivity as well as 90%, 95%, and 99% sensitivity to evaluate specificity. This means that the cutoffs were dynamic, and changed during each iteration of cross-validation. A list of all predicted probability cutoffs may be found in Supplementary Table S3.

### Cross-Validation

To study the models and cutoffs more vigorously, the data set of 568 cases was subjected to 1,000 iterations of 10-fold cross-validation. The folds were generated using stratified sampling through the “caret” package in R^37^. Each model building data set (nine folds) was used to train five algorithms based on the five models, allowing for a more complete understanding of the efficacy of each individual model (AUC, sensitivity and specificity). For the cross-validation data, that is, the single fold of data not included in the model training set, (indicated in this report as the “validation set”), the models created in the model building set were used to predict disease status and were again evaluated on their sensitivity, specificity, and AUC.

### Analysis of 10-Fold Cross Validation Results to Assess Accuracy and Robustness

The cutoffs mentioned above were generated for each model after each bootstrap run on both model building and validation sets. At the end of the 1,000 completed iterations, the performance measures associated with each list of cutoffs were averaged (95% confidence intervals were also calculated) and the medians with ranges were determined. This assessed each model’s accuracy and robustness.

## Electronic supplementary material

Supplementary Information

## References

[1] Ferlay J (2015). Cancer Incidence and Mortality Worldwide: sources, methods, and major patterns in GLOBOCAN 2012. Int J Cancer..

[2] Siegel RL, Miller KD, Jemal A (2017). Cancer statistics, 2017. CA: A Cancer Journal for Clinicians.

[3] El-Serag HB (2012). Epidemiology of Viral Hepatitis and Hepatocellular Carcinoma. Gastroenterology.

[4] Daniele B, Bencivenga A, Megna AS, Tinessa V (2004). Alpha-fetoprotein and ultrasonography screening for hepatocellular carcinoma. Gastroenterology..

[5] Bruix J, Sherman M (2011). Management of hepatocellular carcinoma: an update. Hepatology..

[6] Lin SY (2011). A locked nucleic acid clamp-mediated PCR assay for detection of a p53 codon 249 hotspot mutation in urine. J. Mol. Diagn..

[7] Jain S (2012). Impact of the Location of CpG Methylation within the *GSTP1* Gene on Its Specificity as a DNA Marker for Hepatocellular Carcinoma. PLoS ONE..

[8] Jain S, Wojdacz TK, Su Y-H (2013). Challenges for the application of DNA methylation biomarkers in molecular diagnostic testing for cancer. Expert Rev. of Mol. Diagn..

[9] Su YH, Lin SY, Song W, Jain S (2014). DNA markers in molecular diagnostics for hepatocellular carcinoma. Expert Rev. Mol. Diagn..

[10] Jain S (2015). Differential methylation of the promoter and first exon of the RASSF1A gene in hepatocarcinogenesis. Hepatol. Res..

[11] Hann HW (2017). Detection of urine DNA markers for monitoring recurrent hepatocellular carcinoma. Hepatoma Res..

[12] Chen, D., Jain, S., Su, Y. H. & Song, W. Building Classification Models with Combined Biomarker Tests: Application to Early Detection of Liver Cancer. *J. of Stat. Sci. and App*. (In Press, No. JSSA-E20170424-01) (2017). 10.17265/2328-224X/2017.0506.002 PMC5687830 29152526

[13] Nault JC, Zucman-Rossi J (2014). Genetics of hepatocellular carcinoma: the next generation. J. Hepatol..

[14] Ozen C (2013). Genetics and epigenetics of liver cancer. N. Biotechnol..

[15] Jain S, Singhal S, Lee P, Xu R (2010). Molecular genetics of hepatocellular neoplasia. Am. J. Transl. Res..

[16] Sanyal AJ, Yoon SK, Lencioni R (2010). The etiology of hepatocellular carcinoma and consequences for treatment. Oncologist..

[17] Jia HL (2007). Gene expression profiling reveals potential biomarkers of human hepatocellular carcinoma. Clin. Cancer Res..

[18] Wang M (2009). Novel fucosylated biomarkers for the early detection of hepatocellular carcinoma. Cancer Epidemiol. Biomarkers Prev..

[19] Laxman B (2008). A first-generation multiplex biomarker analysis of urine for the early detection of prostate cancer. Cancer Res..

[20] Li C (2012). Using the k-nearest neighbor algorithm for the classification of lymph node metastasis in gastric cancer. Comput. Math. Methods Med..

[21] Karabatak M (2015). A new classifier for breast cancer detection based on Naïve Bayesian. Measurement..

[22] Dietterich, T. G. Ensemble methods in machine learning. 1–15. (Springer-Verlag, 2000).

[23] Kourou K (2015). Machine learning applications in cancer prognosis and prediction. Comput. Struct. Biotechnol. J..

[24] Kazan H (2016). Modeling Gene Regulation in Liver Hepatocellular Carcinoma with Random Forests. BioMed Res. Int..

[25] Pang H (2006). Pathway analysis using random forests classification and regression. Bioinformatics..

[26] Breiman L (2001). Random forest. Machine Learning.

[27] Wu B (2003). Comparison of statistical methods for classification of ovarian cancer using mass spectrometry data. Bioinformatics.

[28] Cruz JA, Wishart DS (2017). Applications of machine learning in cancer prediction and prognosis. Cancer Inform..

[29] R Core Team R A language and environment for statistical computing. R Foundation for Statistical Computing, Vienna, Austria. https://www.R-project.org/ (2016).

[30] Hosmer, D. W. & Lemeshow, S. Applied Logistic Regression. New York City: John Wiley & Sons, Inc (1989).

[31] Sing T, Sander O, Beerenwinkel N, Lengauer T (2005). ROCR: visualizing classifier performance in R. Bioinformatics..

[32] Breiman, L., Friedman, J. H., Olshen, R. A., Stone, C. J. Classification and Regression Trees. CRC Press; 1984.

[33] Therneau T, Atkinson B, Ripley B (2017). rpart: Recursive Partitioning and Regression Trees. R package version.

[34] Hothorn T, Buehlmann P, Dudoit S, Molinaro A, Van Der Laan M (2006). Survival Ensembles. Biostatistics..

[35] Strobl C, Boulesteix AL, Zeileis A, Hothorn T (2007). Bias in random forest variable importance measures: illustrations, sources and a solution. BMC Bioinformatics..

[36] Strobl C, Boulesteix AL, Kneib T, Augustin T, Zeileis A (2008). Conditional Variable Importance for Random Forests. BMC Bioinformatics..

[37] Kuhn M (2017). caret: Classification and Regression. Training. R package version.

